# Cor a 14, the allergenic 2S albumin from hazelnut, is highly thermostable and resistant to gastrointestinal digestion

**DOI:** 10.1002/mnfr.201500071

**Published:** 2015-08-06

**Authors:** Sabine Pfeifer, Merima Bublin, Pawel Dubiela, Karin Hummel, Judith Wortmann, Gerhard Hofer, Walter Keller, Christian Radauer, Karin Hoffmann‐Sommergruber

**Affiliations:** ^1^ Department of Pathophysiology and Allergy ResearchCenter for PathophysiologyInfectiology and ImmunologyMedical University of ViennaViennaAustria; ^2^ VetCore FacilityUniversity of Veterinary MedicineViennaAustria; ^3^ Institute of Molecular BiosciencesStructural BiologyUniversity of GrazGrazAustria

**Keywords:** Allergen, Cor a 14, Food allergy, Hazelnut (*Corylus avellana*), 2S albumin

## Abstract

**Scope:**

Allergens from nuts frequently induce severe allergic reactions in sensitive individuals. The aim of this study was to elucidate the physicochemical characteristics of natural Cor a 14, the 2S albumin from hazelnut.

**Methods and results:**

Cor a 14 was purified from raw hazelnuts using a combination of precipitation and chromatographic techniques. The protein was analyzed using gel electrophoresis, MS, and far‐UV circular dichroism (CD) analyses. The immunoglobulin E (IgE) binding of native, heat‐treated, and in vitro digested Cor a 14 was studied. We identified two different Cor a 14 isoforms and showed microclipping at the C‐terminus. CD spectra at room temperature showed the typical characteristics of 2S albumins, and temperatures of more than 80°C were required to start unfolding of Cor a 14 demonstrating its high stability to heat treatment. In vitro digestion experiments revealed that Cor a 14 is resistant to proteolytic degradation. Native and heat‐treated protein was recognized by sera from hazelnut allergic patients. However, denaturation of the allergen led to significantly reduced IgE binding.

**Conclusion:**

We identified two different isoforms of Cor a 14 displaying high stability under heating and gastric and duodenal conditions. Data from IgE‐binding experiments revealed the existence of both, linear and conformational epitopes.

**Abbreviations**
CD: circular dichroism
GI: gastrointestinal
IgE: immunoglobulin E
SGF: simulated gastric fluid

## 1 Introduction

In the recent past, the incidence of food allergy has increased. Based on numerous studies, food allergy affects 1–3% of adults and 3–8% of children 1, 2. Tree nuts and peanut account for the majority of severe food allergic reactions. There have been several studies estimating the prevalence of tree nut allergy as being 0.5–1.3%, depending on the study cohort and design 1, 2. Although there are geographic and age‐related variations regarding the severity of symptoms, the overall prevalence of allergic reactions to hazelnut increases 3, 4, 5. In birch endemic areas, hazelnut allergy has mainly been associated with cross‐reactive immunoglobulin E (IgE) to Bet v 1 and Bet v 2, usually causing mild symptoms known as oral allergy syndrome 3, 4. The cross‐reactive allergens in hazelnut are Cor a 1.04 (Bet v 1‐homologue) and Cor a 2 (profilin) 6, 7. More severe and systemic reactions are generally associated with sensitization to the hazelnut nonspecific lipid transfer protein, Cor a 8 8, 9, the 11S legumin‐like protein, Cor a 9 10, 11, 12, the 7S vicilin‐like protein, Cor a 11 13, 14, and the 2S albumin, Cor a 14 11, 12.

2S albumins, belonging to the prolamin superfamily, occur widely in dicotyledonous plants and have been identified as major allergens from many seeds such as Brazil nut, cashew nut, peanut, buckwheat, castor bean, and sesame seed (reviewed in 15). They are structurally homologous proteins with a high content of sulphur‐containing amino acids leading to high nutritional quality. A well‐conserved pattern of cysteine residues resulting in four intramolecular disulfide bonds leads to a well‐conserved structure enriched in α‐helices. The majority of these proteins are proteolytically processed into a small and a large subunit. 2S albumins are encoded by multigene families resulting in numerous isoforms. Additional post‐translational modifications lead to a high degree of heterogeneity. Based on their compact 3D structure these proteins are highly stable to food processing 16, 17 and gastrointestinal (GI) proteolytic degradation 18, 19, 20, 21. This stability is thought to be responsible for the high degree of allergenicity as it enables the intact protein containing active epitopes to reach the gut‐associated lymphoid tissue.

Cor a 14, the 2S albumin from hazelnut has been described as a high risk marker for severe hazelnut allergic phenotype. Masthoff et al. showed that IgE to Cor a 14 had a diagnostic specificity of greater than 90% and accounted for 83% of children and 44% of adults with hazelnut allergy 12. Also a Belgian study demonstrated that IgE to Cor a 14 and Cor a 9 enables to identify almost 90% of preschool and 80% of school‐aged children but only 29% of adults with generalized reactions to hazelnut 11.

Although it has been demonstrated that Cor a 14 is a marker for a severe hazelnut allergic phenotype, only little is known about its physicochemical characteristics. To date the only study is that of Garino et al, who isolated natural Cor a 14 and performed cDNA cloning of Cor a 14 with subsequent characterization of the recombinant protein 22. However, nothing is known about the stability of this important food allergen. Therefore, the aim of this study was to purify natural Cor a 14 isoforms and to elucidate their physicochemical characteristics. Further, we wanted to examine the effect of heat treatment and GI digestion on their capacity to bind specific IgE.

## 2 Materials and methods

### 2.1 Materials

Raw hazelnuts (Beach Flower Back Mix, Turkey) were purchased from a local store. If not stated otherwise chemicals were purchased from SIGMA‐Aldrich (Vienna, Austria). Mono Q Sepharose and the HiLoad 16/60 Superdex 200 prep grade column were obtained from GE Healthcare, Little Chalfont, UK. Natural purified Bos d 5, the allergenic beta‐lactoglobulin from cow's milk was kindly provided by Jean‐Michel Wal and Karine Adel‐Patient, INRA‐Immuno‐Allergie Alimentaire, CEA de Saclay, France. Sera from hazelnut allergic patients were obtained from outpatient clinics and stored at ‐20°C until use. Diagnosis of hazelnut allergy was based on a convincing case history, positive skin prick test and CAP with hazelnut extract. The use of serum samples for this study was approved by the local ethics committee (GS4‐EK‐4/143‐2011). Informed written consent was obtained from all participants.

### 2.2 Purification of natural Cor a 14

Shelled, raw hazelnuts were frozen, ground in a blender and defatted by extraction with hexane. Dried hazelnut flour was extracted with ten volumes of extraction buffer (20 mM sodium acetate, pH 4.5, containing 0.5 M NaCl and 3% polyvinyl polypyrrolidone). Globulins were removed by precipitation with cold methanol (60%, v/v) and prolamins were precipitated with acetone. The prolamin containing fraction was loaded onto a Q Sepharose column with 20 mM Tris‐HCl, pH 7.5 and proteins bound to the column were eluted with increasing concentrations (0–50%) of 1 M NaCl. The chromatogram showed two major peaks (peak 1 and 2) that were further separated by ion‐exchange chromatography using a Mono Q column equilibrated with 20 mM Tris‐HCl, either pH 6.8 (peak 1) or pH 8 (peak 2). Proteins were eluted by a linear gradient of increasing concentrations (0–20%) of 1 M NaCl, resulting in different 2S albumin containing fractions that were then designated Cor a 14 batches 1–5. Batch 14_5, additionally containing high molecular mass proteins, was further purified by gel filtration chromatography (HiLoad 16/60 Superdex 200 prep grade column). RP‐HPLC (Jupiter C5 column, 300 Å, 250 × 10.0 mm, Phenomenex, Aschaffenburg, Germany) was employed as final purification step. Samples were eluted using 0.1% w/v TFA in water as solvent A and 0.085% w/v TFA in water/acetonitrile (10:90 v/v) as solvent B. The column was equilibrated with 10% solvent B and the elution performed as follows: 5 min with 10% solvent B, followed by a linear gradient of 10–35% B within 35 min at 5 mL/min. Proteins were detected by their absorbance at 215 and 280 nm. Protein containing fractions were analyzed by 15% SDS‐PAGE under reducing and nonreducing conditions.

### 2.3 Denaturation by carbamidomethylation

For separation of the subunits, Cor a 14 was dissolved in 500 μL 6 M guanidinium chloride, 0.1 M Tris‐HCl pH 8.4, 1 mM EDTA. After two hours at 37°C, DTT was added to a final concentration of 10 mM and samples were incubated at 60°C for 10 min. After cooling to room temperature, iodoacetamide was added to a final concentration of 200 mM and samples were incubated at room temperature overnight and directly subjected to RP‐HPLC (Jupiter C5 column, 300 Å, 250 × 4.6 mm, Phenomenex, Aschaffenburg, Germany). The program was as follows: 5 min with 0% B, followed by a linear gradient of 0–60% B within 55 min 1 mL/min. Proteins were detected by their absorbance at 215 and 280 nm.

### 2.4 MALDI‐TOF/TOF‐MS and MALDI top‐down sequencing (in‐source decay)

For MALDI‐TOF/TOF peptide identification Cor a 14 samples were digested in solution with Trypsin/LysC‐Mix (Promega, Madison, USA) according to the manufacturers protocol for proteolytically resistant proteins. For improved spectra interpretation N‐terminal peptide derivatization with 4‐sulfophenyl isothiocyanate was performed 23. All mass spectrometric data acquisition was done on a MALDI‐TOF/TOF mass spectrometer (Ultraflex II, Bruker Daltonics, Bremen, Germany).

For intact mass measurements unreduced Cor a 14 and subunits after reduction were spotted with 2,5‐dihydroxybenzoic acid (20 mg/mL in 30% acetonitrile and 70% aqueous TFA 0.1%, 1:1 ratio) onto a ground steel MALDI target plate and measured in reflector mode.

In‐source decay 24 of the small subunits was measured based on a reflector acquisition method with some modifications: mass range of 100–7000 Da and matrix suppression up to 100 Da. Furthermore, detector gain was enhanced to 20×. Up to 6000 shots were acquired per spectrum.

For peak picking and data interpretation flexAnalysis 3.0, Biotools 3.2 and Proteinscape 2.1 (all Bruker software packages) as well as an in‐house Mascot server version 2.3 (Matrix Science, Boston, MA, USA) were used.

Following search parameters were used for TOF/TOF/ISD searches, respectively: database “Cora14 subunits” based on NCBI entry D0PWG2_CORAV; taxonomy all entries; carbamidomethylation on cysteine fixed/variable, other variable modifications valid for both types oxidation on methionine; deamidation on asparagine and glutamine as well as formation of pyroglutamic acid, SPITC for TOF/TOF only; semitrypsin/no enzyme; charge state *z* = +1; MS tolerance 100 ppm; MS/MS tolerance 1 Da; 2 missed cleavages/no missed cleavage; significance threshold *p* < 0.05.

### 2.5 Circular dichroism spectroscopy

Circular dichroism (CD) spectra of native Cor a 14 (0.2 μg/μL in H_2_O) were measured from 190 to 260 nm on a Jasco J‐810 spectropolarimeter (Jasco International Co., Hachioji, Tokyo) at 20°C using a 1 mm path length quartz cell. The effect of heating (2°C/min) was measured at 222 nm. Spectra represent the average of four accumulations collected at 100 nm/min with a 2 s time constant, 0.5 nm resolution, and sensitivity of ±100 mdeg. The secondary structure composition was calculated using the Dichroweb server (program: CDSSTR; reference set: SET 7 Optimized for 190–240 nm) 25.

### 2.6 Simulated gastrointestinal digestion

In vitro gastric (phase I) and duodenal (phase II) digestion of Cor a 14 was performed as described by Moreno et al. 19. Enzymes were purchased from Sigma‐Aldrich: pepsin (P6887), trypsin (T1426), and chymotrypsin (C7762). Briefly, purified Cor a 14 as well as BSA and Bos d 5 as controls were dialyzed against simulated gastric fluid (SGF) 0.15 M NaCl, pH 2.5 and diluted to a final concentration of 0.5 μg/μL, respectively. Pepsin (0.32% in SGF, pH 2.5) was added at a physiological ratio of enzyme/substrate (1:20, w/w) and digestion was performed at 37°C. Aliquots were taken at scheduled time points (0, 2, 5, 15, 30, 60, and 120 min) and the reaction was stopped by increasing the pH to 7.5.

Following gastric digestion, in vitro duodenal digestion was prepared by adjusting the pH to 6.5 and adding a bile salt mixture containing equimolar quantities (7.4 mM) of taurocholic acid sodium salt and glycodeoxycholic acid, 9.2 mM CaCl_2_ and 25 mM Bis‐Tris, of pH 6.5. Finally, trypsin and chymotrypsin were added at physiological ratios of enzyme/substrate 1:400 and 1:100, w:w, respectively. The digestion was performed at 37°C with shaking and aliquots were taken after 2, 5, 15, 30, 60, and 120 min. Subsequently, samples were analyzed by 15% SDS‐PAGE and immunoblotting using a rabbit antiserum raised against natural, purified Cor a 14. To demonstrate the functionality of the assay, the digestion was performed for single proteins as well as in a mixed assay format. While the mixed assay was used for the SDS‐PAGE analysis and the immunoblot, single Cor a 14 digestion was subsequently used for the IgE ELISA experiments.

### 2.7 IgE ELISA

Microtiter plates (Nunc, Roskilde, Denmark) were coated with 0.5 μg protein (Cor a 14 native, reduced, and digested, respectively) per well and blocked with Tris‐buffered saline containing 0.5% v/v Tween 20 (TBST) and 3% w/v BSA. Sera from allergic patients and non‐allergic control subjects were diluted 1:10 in TBST and applied onto the plates followed by an overnight incubation at 4°C. Bound IgE was detected by incubation with 1:1000 diluted alkaline phosphatase‐conjugated mouse anti‐human IgE antibody (BD BioSciences, Heidelberg, Germany) for 2 h at room temperature, and color development was performed by using disodium p‐nitrophenyl phosphate substrate tablets. OD was measured at 405 nm, and the mean value of the negative controls was subtracted. The Wilcoxon signed rank test was used for comparison of IgE binding to native with heated, reduced, and digested Cor a 14. *p*‐values below 0.05 were considered statistically significant. Analyses were performed with GraphPad Prism software (GraphPad Software, La Jolla, CA, USA). All sera were tested in duplicates.

## 3 Results

### 3.1 Purification and characterization of natural Cor a 14

**Figure 1:**
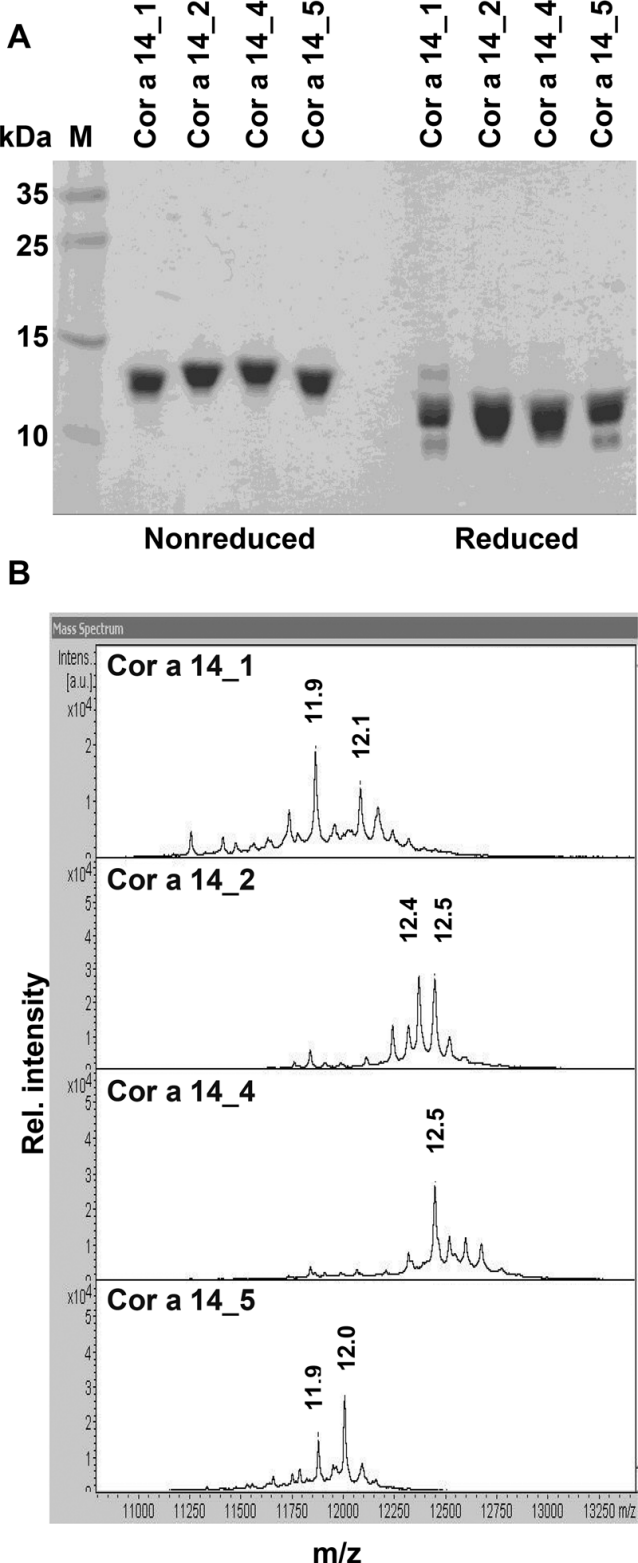
Purification of natural Cor a 14. (A) 15% Coomassie stained SDS‐PAGE under nonreducing and reducing conditions. (B) Mass spectra of the different Cor a 14 batches (nonreducing).

Cor a 14 was purified from hazelnut extract with a yield of 100 mg, corresponding to 0.6% of total protein content obtained from 100 g hazelnuts. Separation from the prolamin fraction by anion exchange chromatography resulted in five different batches (Cor a 14_1–5) eluting at different salt concentrations. Coomassie stained SDS‐PAGE analysis revealed that, although slight differences in size were visible, all batches were highly enriched in proteins with a molecular mass of ∼12 kDa. Since the yield of batch 3 was low, further experiments were performed with batches 1, 2, 4, and 5. Upon reduction, proteins from all batches gave rise to two bands, one of about 8 kDa corresponding to the large subunit and a smaller band that was not completely resolved in batches 2 and 4 corresponding to the small subunit. It became visible that size differences of the native protein are due to size differences of the small subunit (Fig. 1A). MALDI‐TOF‐MS analysis yielded masses between 12 kDa (14_1 and 5) and 12.5 kDa (14_2 and 4) for the native proteins (Fig. 1B), which after reduction gave rise to two bands of M_r_ ∼3800–4500 and ∼8200 likely to correspond to the small and large subunits of the 2S albumin, respectively.

**Figure 2:**
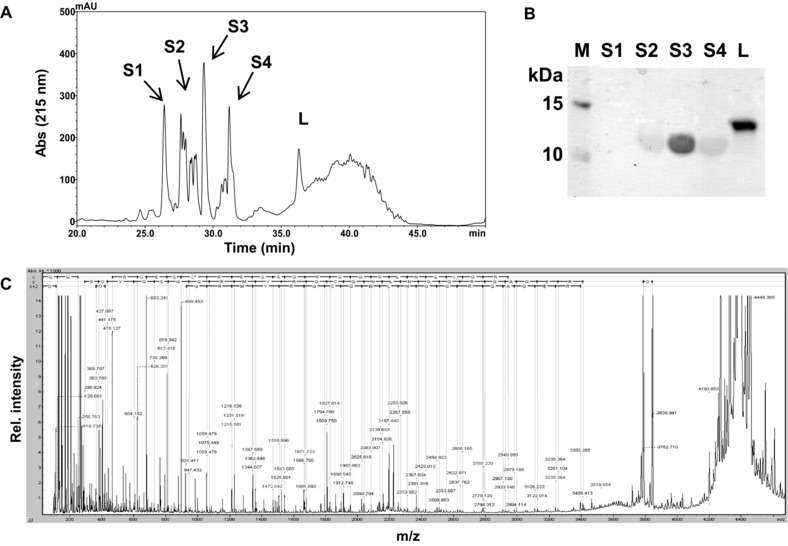
Physicochemical characterization of purified Cor a 14. (A) RP‐HPLC chromatogram of reduced/alkylated Cor a 14 (batch 2) (S, small subunits; L, large subunit) and (B) the respective SDS‐PAGE. (C) Representative in‐source decay spectrum of the small subunit.

To examine the subunits in more detail, RP‐HPLC of reduced and alkylated Cor a 14 was performed. After reduction, all batches gave rise to several peaks. SDS‐PAGE analysis identified the fractions as one large and several small subunits (Fig. 2A and B). MALDI top‐down proteomic identification of the separated subunits (Fig. 2C) yielded the complete sequences of the differently eluting small subunits which are summarized in Table 1. Two isoforms of the small subunit (R/S at position 10 of the mature protein) were identified, both existing as a heterogeneous mixture due to posttranslational modifications. Identified modifications were clipping at the C‐terminus, cyclization of the N‐terminal glutamine to pyroglutamic acid, and carbamidomethylation of cysteines 9 and/or 22 (due to alkylation with iodoacetamide). The most abundant form of the small subunit, in accordance with the published cDNA sequence (UniProt: D0PWG2), was Pyro‐QQGRRGESCREQAQRQQNLNQCQRYMRQQSQYGSYD with arginine at position 10, carbamidomethylation of aa 9 and 22, and a molecular mass of 4505 Da.

**Table 1:** Amino acid sequences and modifications of the small subunits of Cor a 14 identified by in‐source decay

Batch	Mass	Mascot score	Sequence
1	3862.8	111	a QQGRRGES C REQAQRQQNLNQCQRYMRQQSQ
1	3919.8	133	a QQGRRGES C REQAQRQQNLNQ C QRYMRQQSQ
1	4169.9	96	a QQGRRGES C REQAQRQQNLNQCQRYMRQQSQYGS
1	4226.9	106	a QQGRRGES C REQAQRQQNLNQ C QRYMRQQSQYGS
2	4378.9	87	a QQGRRGES C **S** EQAQRQQNLNQCQRYMRQQSQYGSYD
2	4435.9	111	a QQGRRGES C **S** EQAQRQQNLNQ C QRYMRQQSQYGSYD
2	4448.4	87	a QQGRRGES C REQAQRQQNLNQCQRYMRQQSQYGSYD
2	4505.0	115	a QQGRRGES C REQAQRQQNLNQ C QRYMRQQSQYGSYD
4	4448.0	100	a QQGRRGES C REQAQRQQNLNQCQRYMRQQSQYGSYD
4	4505.0	139	a QQGRRGES C REQAQRQQNLNQ C QRYMRQQSQYGSYD
5	3793.7	90	a QQGRRGES C **S** EQAQRQQNLNQCQRYMRQQSQ
5	4013.8	81	a QQGRRGES C **S** EQAQRQQNLNQCQRYMRQQSQYG
5	4070.8	146	a QQGRRGES C **S** EQAQRQQNLNQ C QRYMRQQSQYG
5	4157.8	135	a QQGRRGES C **S** EQAQRQQNLNQ C QRYMRQQSQYGS

a Cyclization of glutamine to pyroglutamic acid.

Underlined, carbamidomethylation of cysteine. Bold, different residues in isoform 2.

Whereas several variants of the small subunit were identified by RP‐HPLC, only one signal that corresponds to the large subunit was detected (Fig. 2A and B). MALDI‐MS revealed that the large subunits of all batches had identical molecular masses (data not shown). Tryptic digest followed by MALDI‐TOF/TOF peptide analysis confirmed the published sequence with a coverage of up to 100% (batch 2) and the C‐terminal cleavage of three amino acids (ARF). No additional variants were identified.

### 3.2 Secondary structure and thermostability

**Figure 3:**
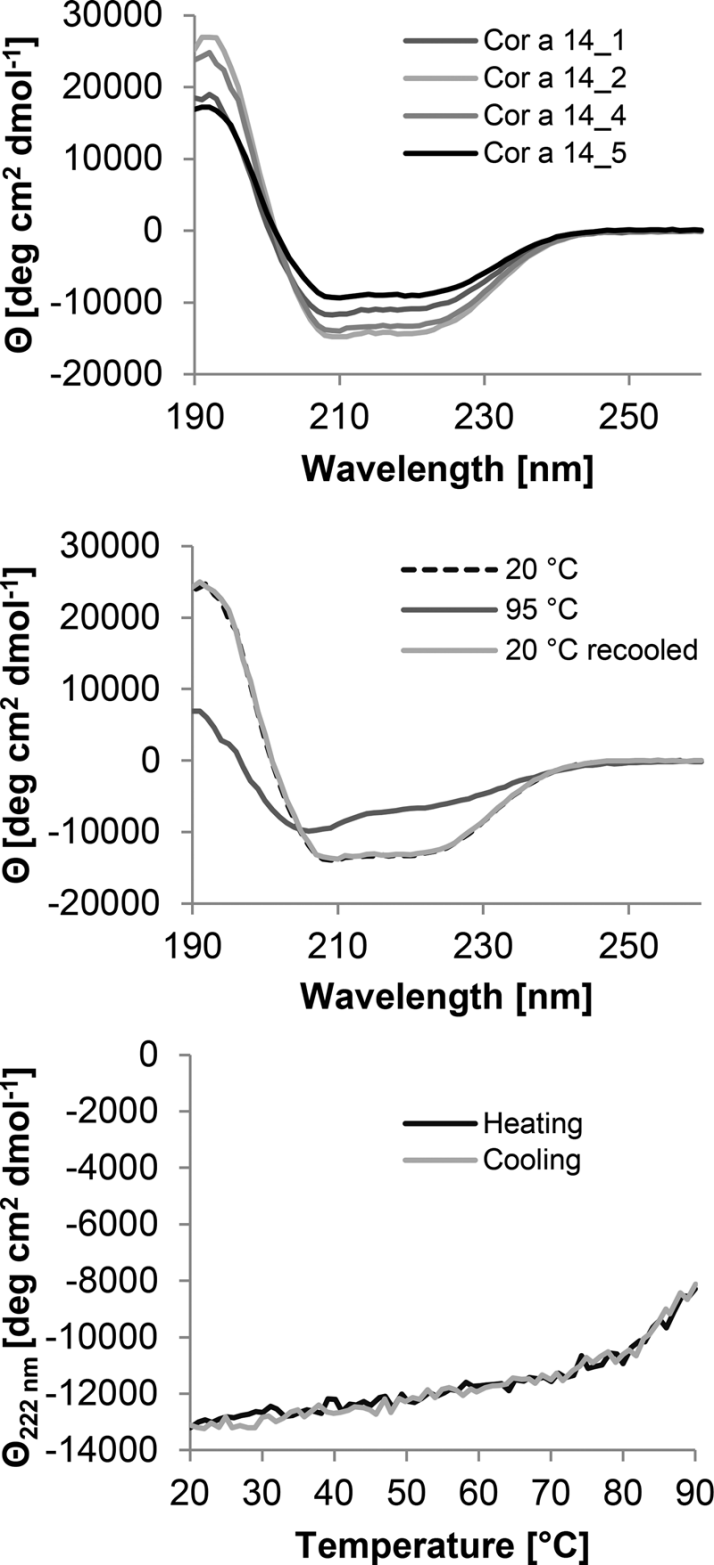
Secondary structure analysis of purified Cor a 14. Far‐UV CD spectra of Cor a 14. (A) Overlay of the spectra of the different batches at room temperature at neutral pH. (B) Change in molar ellipticity at 222 nm during heating and cooling (batch 4). (C) Spectra of Cor a 14 (batch 4) at room temperature (light gray line), after heating to 95°C (dotted line), and after recooling (dashed line) at neutral pH.

The overall secondary structure composition as well as investigation of heat stability was examined by CD spectroscopy. CD spectra of the native protein showed the typical characteristics of 2S albumins with negative extremes at 208 nm and 222 nm and a positive maximum at 193 nm, representative of high α‐helical content. All batches showed almost similar spectra, corresponding to high α‐helical contents in the range of 58–74% of the overall structure (Fig. 3A). Results of the Dichroweb analysis of Cor a 14 are summarized in Table 2.

**Table 2:** Secondary structure composition of Cor a 14 (batches 1–5) from the Dichroweb analysis of CD spectra

Batch	Helix	Strand	Turn	Unordered
1	76%	6%	11%	6%
2	67%	12%	9%	11%
4	67%	15%	10%	9%
5	74%	6%	6%	16%

The effect of heat treatment on the secondary structure of Cor a 14 was examined. Temperatures above 80°C were required to start unfolding of Cor a 14 (Fig. 3B). At 95°C the protein was partly unfolded, but still showed the characteristic features of α‐helical proteins. After recooling to 20°C the CD signal was fully recovered demonstrating that denaturation was completely reversible (Fig. 3C).

### 3.3 In vitro digestibility of Cor a 14

**Figure 4:**
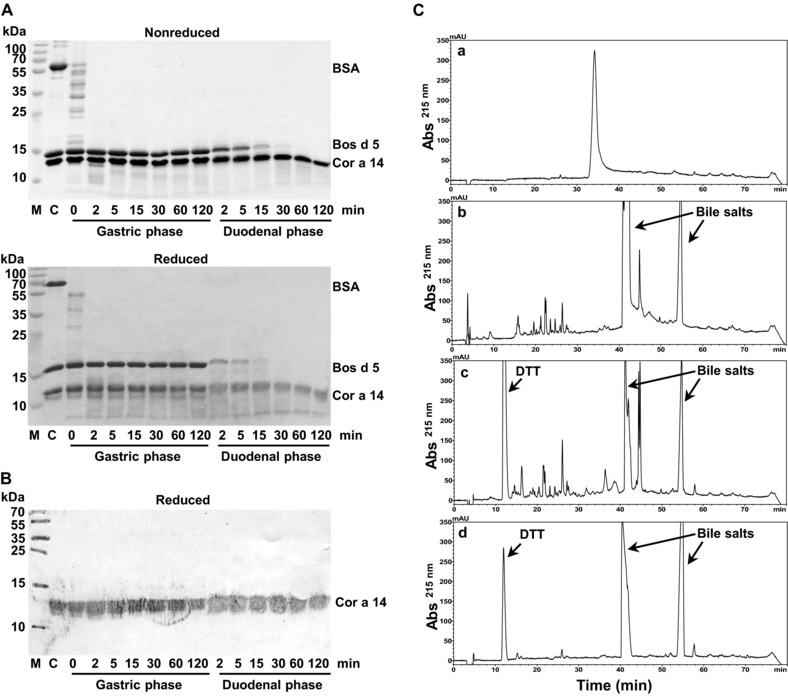
Effect of simulated gastric and duodenal digestion on purified Cor a 14 (batch 4). (A) SDS‐PAGE of digested samples after the indicated times of gastric and duodenal digestion, respectively. BSA and Bos d 5 were used as controls for gastric and duodenal digestion, respectively. C: a mixture of untreated Cor a 14, BSA and Bos d 5 was used as negative control. (B) Western blot analysis of digested samples at reducing conditions using a polyclonal rabbit anti‐Cor a 14 antibody. (C) RP‐HPLC chromatograms of (a) native, (b) digested, (c) digested/reduced Cor a 14 (batch 4), and (d) a control without Cor a 14 at 215 nm.

As demonstrated by SDS‐PAGE, Cor a 14 was resistant to gastric digestion. Even after two hours of pepsinolysis, one prominent band was visible corresponding to the intact protein. BSA, which is known to be degraded by pepsin, was used as positive control and degraded immediately after addition of the enzyme. After two hours of gastric digestion, pH was raised to 6.5, and the intestinal enzymes trypsin and chymotrypsin were added. Bos d 5 was used as positive control for duodenal digestion. In line with earlier studies, Bos d 5 was stable to gastric conditions but rapidly degraded during duodenal digestion. Cor a 14 however remained intact during the whole process, indicating that the purified allergen was completely resistant to proteolytic degradation (Fig. 4A). Using Cor a 14‐specific antibodies, Western blot analysis of the digested protein showed that Cor a 14 appeared as two bands (Fig. 4B). RP‐HPLC analysis revealed that native Cor a 14 eluted after 34 minutes. After gastric and duodenal digestion the retention time and the shape of the main peak changed and some additional peaks (corresponding to peptide fragments) appeared (Fig. 4C). This demonstrates that although limited proteolysis occurred, the small and large subunit mainly remained intact and disulphide‐linked.

### 3.4 IgE binding

**Figure 5:**
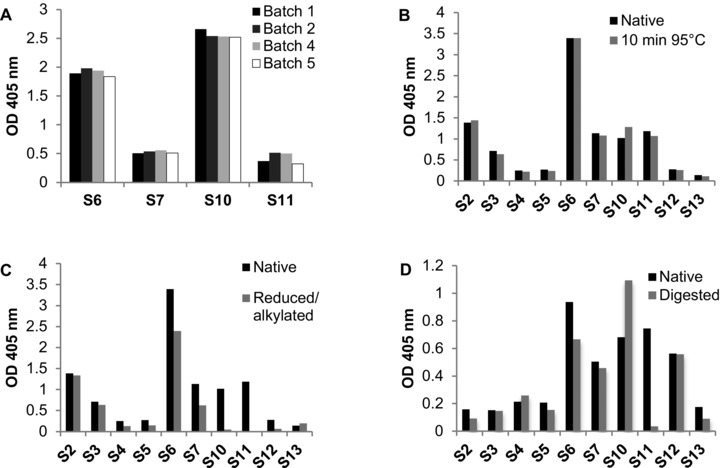
IgE binding of sera from hazelnut allergic patients to Cor a 14. (A) Different batches (1, 2, 4, and 5) of native Cor a 14, (B) heated, (C) reduced and alkylated (R/A), and (D) digested Cor a 14 were tested for their IgE‐binding capacities.

All batches (Cor a 14_1–5) were recognized by specific IgE antibodies derived from allergic patients’ sera with comparable intensity (Fig. 5A). Next, we assessed the IgE‐binding capacity of native versus heat treated Cor a 14 (10 min 95°C). Although slight changes were visible (Fig. 5B), heating of Cor a 14 did not result in overall significant changes of its IgE‐binding capacity (*p* = 0.11; medians 0.84 and 0.77).

Denaturation of Cor a 14 by reduction and alkylation resulted in a significant (*p* = 0.002) decrease of IgE binding (median OD values 0.84 and 0.05). After reduction, 5 of 10 tested sera (S4, S5, S10–S12) did not recognize reduced Cor a 14, while the samples S2, S3, S6, S7 showed decreased IgE binding (Fig. 5C). Serum 13 even displayed slightly higher IgE binding to reduced and alkylated Cor a 14.

In vitro GI digestion of Cor a 14 affected the IgE‐binding activity. Some of the tested sera (S2, S5, S6, S7, S11, and S13) showed reduced IgE binding when compared with untreated protein. Interestingly, 2 sera (S4 and S10) even showed a marked increase (20 and 61%, respectively) in binding to the digested allergen (Fig. 5D). IgE binding of two sera (S3 and S12) was unchanged.

## 4 Discussion

Recent studies have shown that sensitization to Cor a 14 is highly specific for patients with objective allergic symptoms to hazelnut and that it can be used as a risk marker for severe hazelnut allergy in children and adults 11, 12. Although it became apparent that the 2S albumin from hazelnut is an important allergen, little information about its physicochemical properties is available 22.

In this study, we isolated natural Cor a 14, in order to elucidate its physicochemical and IgE‐binding characteristics. We showed that it is processed and presents as two isoforms that differ in only a single amino acid at position ten (R/S) of the small subunit of the mature protein. This low number of isoforms is in contrast to what is known from many other members of this protein family. Generally, 2S albumins are encoded by multigene families encoding several isoforms and thus creating a high level of polymorphism 26, 27. For Ber e 1 six different isoforms were identified 28, 29, 30, while sunflower 2S albumins can be separated into up to 13 components 31, 32. On the other hand Ses i 1 was shown to consist of a single isoform 20.

MALDI top‐down sequencing revealed common N‐termini for all samples analyzed starting at positions 39 and 78 of the precursor sequence for the small and large subunits, respectively. The mass data further indicated that cyclization of the N‐terminal Gln to pyroglutamic acid occurred, a feature common for numerous 2S albumins 29, 30, 33. Interestingly, these results differ from that published by Garino et al. 22, who reported N‐terminal sequences of the large and small subunits shifted by 4 and 42 residues towards the C‐terminus, respectively. Apparently, these sequences were obtained from internal peptides after tryptic digest.

Detailed analysis further revealed that truncations of the C‐terminus of the small subunit occurs, giving rise to a high level of micro‐heterogeneity (Table 1). Such micro‐heterogeneity of C‐termini has been described for many 2S albumins, including those from castor bean, rapeseed, and Brazil nut 30, 34, 35. In some cases this had an impact on the IgE‐binding activity. In contrast, we did not detect heterogeneity of the N‐terminus of Cor a 14.

In summary, we obtained several purified Cor a 14 batches consisting of a shared identical large subunit together with different small subunits. Batches 1 and 4 contained the small subunit containing aa R at position 10 that is in accordance with the published sequence (UniProt: D0PWG2). In contrast, batch 5 contained the small subunit with aa S at position 10 representing a new isoform, whereas batch 2 was a mixture of both small subunit isoforms. In addition, micro‐heterogeneity of the C‐terminus of the small subunits was responsible for their slightly different behavior during the purification process. For the large subunit, we confirmed the sequence obtained from the database with a coverage of 100% and could further show the C‐terminal cleavage of three amino acids. No additional variants were identified.

We showed that Cor a 14 remained intact during the whole process of in vitro GI digestion. Although it still remains unclear what makes an allergen an allergen, properties preserving its structure, such as stability to low pH, bile salts, and proteolytic degradation, is a fundamental feature of a protein to survive the transport through the GI tract and reach the gut‐associated lymphoid tissue. Several 2S albumins have been shown to be highly resistant to GI digestion retaining their compact structure 19, 20, 36.

Importantly, no differences in IgE‐binding capacity of sera from hazelnut allergic patients were detected between the different batches. IgE‐binding experiments using digested Cor a 14 demonstrated that it kept its capacity to bind specific IgE and thus its allergenic potential. In some cases we observed even considerably increased IgE binding. This observation could be explained by a slight change of the conformation and thus a better accessibility of cryptic epitopes. Only one serum did not recognize Cor a 14 after GI digestion. Reduction of the allergen led to a complete loss of IgE binding in 50% of the tested sera, supporting data from other studies in which conformational epitopes have been described 37, 38. The remaining tested sera showed reduced immunoreactivity as compared with the native protein. In these cases the IgE response seems to be directed against both conformational and linear epitopes.

Heat treatment did not lead to significant changes in IgE‐binding capacity indicating that both linear and most of the conformational epitopes were preserved. In accordance with this finding, CD measurement of Cor a 14 revealed that the protein retained most of its secondary structure after heating and completely refolded after cooling. This was also shown for other 2S albumins 37, 38.

In summary, we purified and characterized several batches of natural Cor a 14. In contrast to other 2S albumins, we found limited heterogeneity of the mature protein without notable differences concerning IgE‐binding properties. However, a detailed analysis of the intact N‐ and C‐terminus of a mature protein as well as identification of putative isoforms and their respective allergenic activity is mandatory before selecting the most appropriate sequence/s for recombinant production suitable for component resolved diagnosis.
